# *In Vitro* Effects of Two Topical Varnish Materials and Er:YAG Laser Irradiation on Enamel Demineralization around Orthodontic Brackets

**DOI:** 10.1155/2014/490503

**Published:** 2014-05-27

**Authors:** Feyza Ulkur, Elif Sungurtekin Ekçi, Didem Nalbantgil, Nuket Sandalli

**Affiliations:** ^1^Department of Orthodontics, Faculty of Dentistry, Yeditepe University, Bagdat Caddesi No. 238, Goztepe, Kadikoy, 34728 Istanbul, Turkey; ^2^Department of Paediatric Dentistry, Faculty of Dentistry, Yeditepe University, Bagdat Caddesi No. 238, Goztepe, Kadikoy, 34728 Istanbul, Turkey

## Abstract

The aim of this *in vitro* was to evaluate the effects of tricalcium phosphate (TCP) and amorphous calcium phosphate (ACP) containing varnish materials and Er:YAG laser irradiation on enamel demineralization around orthodontic brackets. Forty extracted human premolar teeth were randomly divided into four treatment groups (i.e., 10 in each group): (1) 5% NaF-ACP varnish, (2) 5% NaF-TCP varnish, (3) Er:YAG laser, and (4) control (no treatment). Er:YAG laser was operated at a wavelength of 2.94 *μ*m and the energy output was 80 mJ per pulse; a pulse duration of 200 *μ*sec and and a frequency of 2 Hz were used with water cooling. All samples were then put into pH cycles. Surface microhardness values and representative SEM images were assessed. Surface microhardness values were evaluated using Kruskal-Wallis and Mann-Whitney *U* tests. The results revealed that demineralization was significantly lower in the TCP and ACP varnish groups, whereas mean surface microhardness values of the TCP varnish were found higher than the ACP (*P* < 0.05). TCP and ACP varnish materials were found effective for reducing enamel demineralization around orthodontic brackets. Use of Er:YAG laser irradiation as described in this study for inhibition of demineralization was found not satisfactory.

## 1. Introduction

Despite the favorable aesthetic and functional outcomes of orthodontic treatment with fixed appliances, negative consequences might be observed on both periodontal tissues and tooth surfaces, particularly in young patients who are not able to maintain adequate oral hygiene during treatment [1]. When oral hygiene is less than favorable, demineralization areas called “white spots” are frequently seen around orthodontic brackets; these spots are characterized by their opacity compared to healthy enamel [2]. These lesions are the first clinical signs of demineralization and are formed by the mineral loss from the enamel that is found in a cycle of demineralization and remineralization in the oral cavity [3].

Besides providing optimum oral hygiene, the risk of enamel demineralization can be prevented or reduced with various conventional methods including the application of remineralizing agents and different forms of fluoride treatments as well as contemporary treatments like laser irradiation. The most frequent method used in clinical practice today is the application of fluoride agents in various forms, which is a proven approach for promoting enamel remineralization [4–6]. Recently, more advanced fluoride varnishes with added calcium and phosphate ions have been developed to supplement the amounts of these ions in saliva and enhance remineralization by fluoride [7]. For example, casein phosphopeptide-amorphous calcium phosphate (CPP-ACP) is reported to have remineralizing effects because of its calcium and phosphate ion content [8, 9]. Calcium and phosphate ions released from ACP materials are delivered over the enamel surface where they form a hydroxyl apatite-like structure [10]. Another development is tricalcium phosphate (TCP), which is a new hybrid material created with a milling technique that fuses beta TCP with simple organic materials to create a functionalized TCP ingredient. When TCP comes into contact with the tooth surface and is moistened with saliva, the protective barrier breaks down making calcium, phosphate, and fluoride ions available to the tooth [11].

Laser irradiation is among the new techniques that might be promising in this field of practice. Recent research has shown that Er:YAG laser can produce positive effects on the increase of enamel acid resistance [12, 13]. In a study by Hsu et al. [14], by employing low-energy laser irradiation, 90% reduction in enamel demineralization was achieved. This study suggests that melting enamel may not be necessary for laser induced caries prevention (LICP); the results also pointed out that the inhibition of enamel diffusion through the modification of the organic matrix might be one of the major mechanisms involved in LICP. The modification and preservation of the organic matrix by low level laser irradiation obliterate the diffusion channels in enamel by causing the effect just opposite to high-energy laser irradiation that melts hydroxyapatite [15].

To date, there are no* in vitro* studies comparing the effects of these conventional and contemporary methods on enamel demineralization around orthodontic brackets. Therefore, the aim of this* in vitro* study was to evaluate the effects of tricalcium phosphate and amorphous calcium phosphate containing varnish materials and Er:YAG laser irradiation on enamel demineralization around orthodontic brackets.

## 2. Methods

This study was conducted using a parallel group design with three experimental groups and a control group. Block randomized selection was used when groups were allocated. Forty human premolar teeth extracted for orthodontic reasons with no active or initial caries lesions with hypoplastic areas, developmental defects, and staining or enamel defects were chosen. Large irregularities of enamel surface and the teeth that had pretreatment after extraction with a chemical agent such as alcohol, formalin, and hydrogen peroxide were excluded. Extracted teeth were stored in 0.1% thymol solution in a refrigerator for no longer than one month until the study started. Before bonding procedures, the teeth were cleaned with a scaler to remove calculus and tissue remnants, and the surfaces were polished with a nonfluoridated pumice and washed with deionized water.

Buccal surface of each tooth was isolated with an adhesive tape, which was cut similar to the bracket base by a hole puncher to standardize and limit the enamel area exposed to the etching and bonding procedures. After the isolation procedure, buccal tooth surfaces were prepared with 37% phosphoric acid gel (3M Dental Products; St Paul, MN, USA) for 20 s, washed for 15 s, and dried with air for 15 s subsequently. Forty brackets (Dyna-Lock series, 100-gauge mesh, 3M Unitek, USA) were bonded on buccal surfaces after Transbond XT primer (3M Unitek; Monrovia, CA, USA) was applied to each tooth and Transbond XT paste (3M Unitek; Monrovia, CA, USA) was used. The bonding agents were cured with a light emitting diode (Mectron Starlight *p* ^*S*^) with a wavelength of 440–480 nm for 20 s. Following the removal of the isolation tapes, the teeth were isolated with two layers of nail varnish by leaving 2 × 2 mm of intact space around the bracket. Teeth were then randomly assigned to the four study groups. Enamel Pro Varnish (5% sodium fluoride varnish-ACP formula, Premier Dental Products Company, PA, USA) (ACP), Clinpro White Varnish (tricalcium phosphate, 3M ESPE, Seefeld, Germany) (TCP), and Er:YAG laser irradiation (Hoya, VersaWaveTM, Tokyo, Japan) were applied to the experimental groups. The fourth group was set as negative control and no treatment was applied. Agents were left undisturbed for five minutes on the tooth surfaces.

Er:YAG laser was operated at a wavelength of 2.94 *μ*m with a round head application tip of 1 mm diameter. The energy output was 80 mJ per pulse, and a pulse duration of 200 *μ*s and pulse frequency of 2 Hz were used with water cooling (5 mL/min). The laser beam was applied for 10 s in noncontact, focused mode at a perpendicular distance of 4 mm.

Following treatment, all samples were then put into pH cycles for 14 days through a daily procedure of de- and re-mineralization. Each specimen was immersed individually in a 60 mL of demineralization solution that contained 2.0 mmol/L calcium, 2.0 mmol/L phosphates, and 75 mmol/L acetate buffers at pH 4.3 for 6 hours. Once removed from the demineralization solution, the specimens were rinsed with deionized water and then immersed individually in 40 mL of a remineralization solution. For the remineralization, the solution contained 1.5 mmol/L calcium, 0.9 mmol/L phosphates, 150 mmol/L potassium chloride, and 20 mmol/L cacodylate buffers at pH 7.0 for 17 hours. As proposed by Hu and Featherstone [16], the specimens were stored at 37°C to stimulate body temperature. After drying for 24 h, some representative specimens were examined under scanning electron microscope (SEM). For this purpose, the specimens were gently air dried and dehydrated with alcohol. All specimens were placed on carbon disc and coated with a 15 nm thick gold layer using a Baltec SDC 005 sputter coater. SEM images at 1000x and 2000x magnification were obtained by using a Carl Zeiss Evo-40 instrument under high vacuum at high potential (i.e., 10 kV).

**Figure 1:**
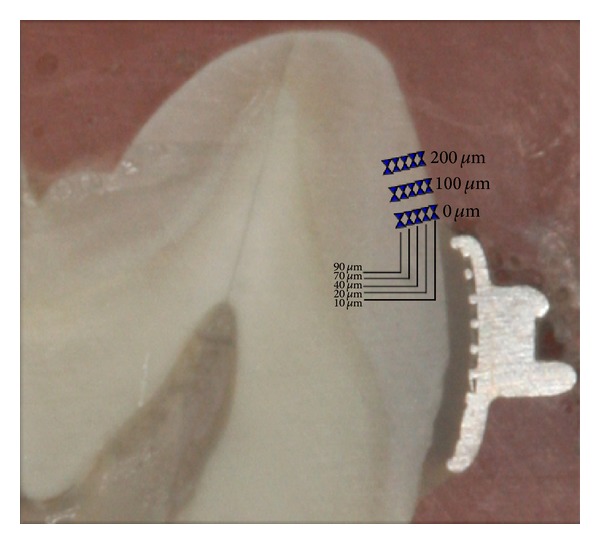
Positions and depths of indentations.

Subsequent to the pH cycling procedure, teeth were sectioned. The roots were removed 2 mm apically to the cementoenamel junction and the crowns were hemisectioned vertically into mesial and distal halves with a 15 HC wafering blade on an Isomet low speed saw (Buehler, Lake Bluff, IL, USA) directly through the slot of the bracket, leaving a gingival and an incisal portion. Each half was embedded in self-curing EpoKwick epoxy resin (Buehler, Germany), leaving the cut face exposed. These half crown sections were then polished with 320, 600, and 1200 grit abrasive paper discs sequentially. The polishing was finalized with a 1 *μ*m diamond spray and polishing cloth disc (Buehler, Germany). The prepared specimens were evaluated quantitatively by cross-sectional microhardness tester (Micromet 5114, Buehler, Lake Bluff, IL, USA) fitted with a Vickers diamond; testing was done by one operator who was blinded from group allocations. Surface microhardness analyses were carried out under a 200 g load for 5 s. On each sample, a total of 15 indentations were made at 3 regions and 5 depths per region. The selected regions were the edge of the bracket base (0 *μ*m) and 100 and 200 *μ*m coronally from the edge of the bracket base. The depths of microhardness were marked at 10, 20, 40, 70, and 90 *μ*m from the outer surface of enamel, as shown in Figure 1. Microhardness values measured from two halves of a crown were averaged.

The data analyses were performed by the Statistical Package for Social Sciences (SPSS, Version 17.0, SPSS, Inc.; Chicago, IL, USA). Data were evaluated using Kruskal-Wallis test. Pairwise comparisons were performed using Mann-Whitney* U* test with Bonferroni correction (number of comparisons = 6). The power analysis showed that 40 pairs of extracted teeth were needed to achieve 84% power to detect differences between the groups at a statistical significance level of *P* < 0.05.

## 3. Results

Descriptive statistics and multiple comparisons of surface microhardness values of specimens are given in Table 1. In general, surface hardness values of the TCP group are found to be the highest followed by ACP and Er:YAG laser groups, whereas control group was the lowest. Surface microhardness values of all groups showed statistically significant differences at all measurement depths (*P* < 0.05).

The pairwise comparison test did not reveal any significant differences between control and Er:YAG laser groups for any of the indentations (*P* > 0.05). On the other hand, surface microhardness values of Er:YAG group were significantly lower than that of TCP group in every indentation except for 0 *μ*m distance and 10 *μ*m depth (*P* = 0.013) (Table 2). The difference between control and TCP groups was also statistically significant in all indentations.

The surface microhardness values of the ACP group were significantly different from control and Er:YAG groups, but these values were not as substantial as those of the TCP group. Significant differences were only found at 0 *μ*m distance in 40 and 90 *μ*m depths (*P* = 0.008 and *P* = 0.002, resp.) between ACP and Er:YAG groups, whereas these were seen at 0 *μ*m distance in 20, 40, 70, and 90 *μ*m depths (*P* = 0.008, *P* = 0.003, *P* = 0.006, and *P* = 0.003, resp.) and at 200 *μ*m distance in 70 and 90 *μ*m depths (*P* = 0.004,  *P* = 0.005, resp.) between ACP and control groups (Table 2).

Regarding the two varnish groups, a significant difference was observed only at 0 *μ*m in 20 *μ*m depth (*P* = 0.005). However, it can be assumed that the differences were becoming more apparent at the indentations when measured further than the bracket.

**Figure 2:**
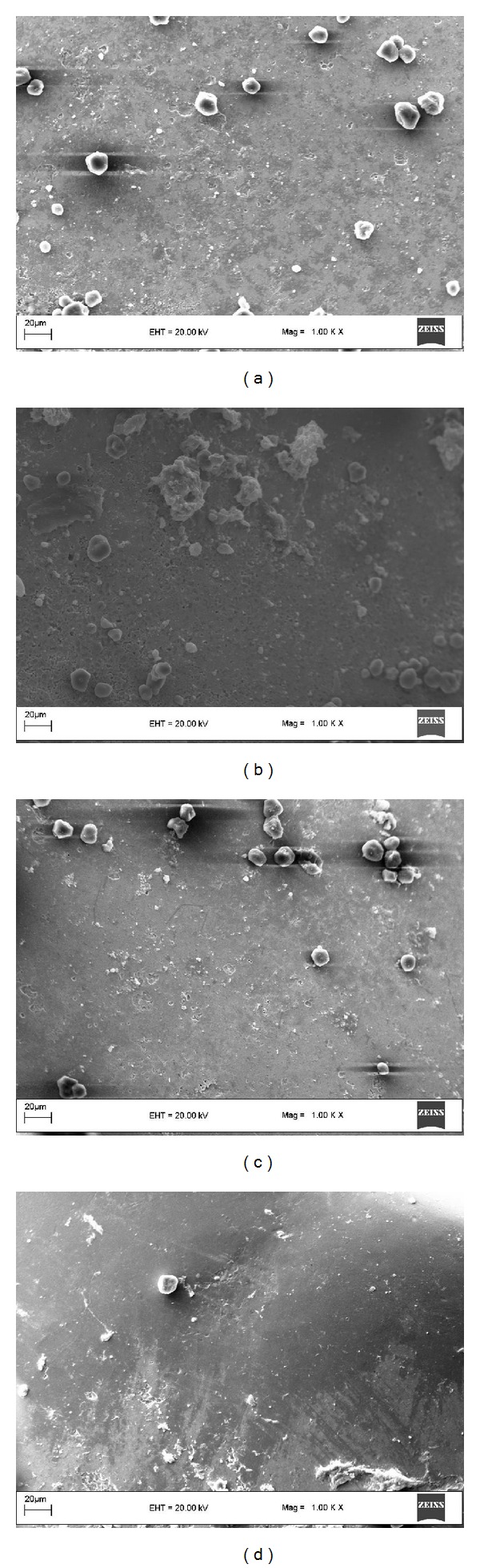
SEM images (1000x magnification) of the specimens from (a) Er:YAG laser, (b) control, (c) ACP, and (d) TCP.

**Figure 3:**
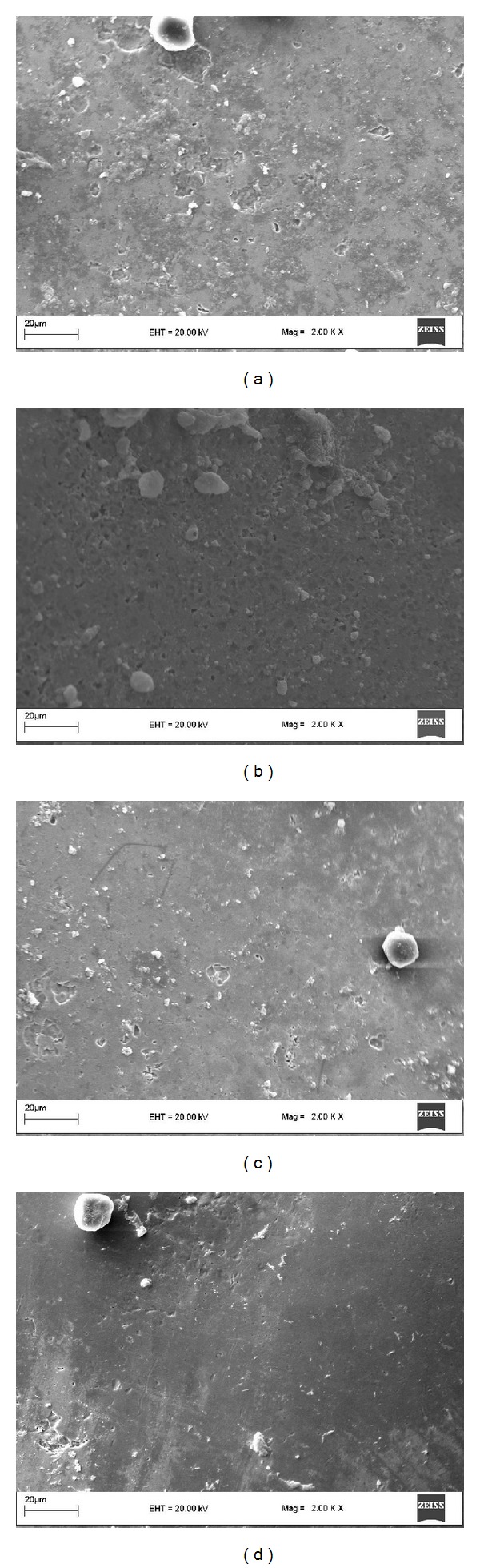
SEM images (2000x magnification) of the specimens from (a) Er:YAG laser, (b) control, (c) ACP, and (d) TCP.

SEM images magnified by 1000x magnification revealed that enamel surfaces were smooth in the TCP and the ACP groups, whereas craters were formed on the enamel surfaces in other groups (Figure 2). Likewise, at 2000x magnification, the TCP and the ACP groups exhibited similar smooth enamel surfaces. On the other hand, Er:YAG laser and control groups showed increasing amount of craters, respectively (Figure 3).

## 4. Discussion

During orthodontic treatment, the main etiological factors of enamel demineralization are the bacterial plaque, diet of the patient, and mineral content of the enamel [17]. It is evident that reduction of demineralization can be accomplished by removing or lessening the effects of these causes. As methods that focus solely on patient compliance have not been completely successful, [16, 18] studies that focus on other approaches have become more important.

The role of calcium phosphate in decreasing the incidence of enamel demineralization has been extensively studied. In the present study, the effects of low level Er:YAG laser irradiation and two different calcium phosphates containing fluoride varnishes on enamel demineralization around brackets were compared. To reach this aim, the mineral loss was evaluated with Vickers microhardness testing, which is a well-accepted method. The results revealed that both calcium phosphates containing fluoride varnishes were effective against demineralization regardless of the depth of measurement, whereas Er:YAG laser irradiation did not show a remarkable effect. Previously, Reynolds [19] reported that remineralization agents containing amorphous calcium phosphate were found to be effective in the treatment of early carious lesions. Recently Uysal et al. [20] using both* in vivo* and* in vitro* tests showed similar results to the present study in terms of the influence of amorphous calcium phosphate varnish; this study stated that the demineralization effect of ACP could be beneficial.

There is an increasing level of research focusing on how calcium and phosphate supplementation of fluoride treatment can enhance fluoride uptake [21, 22]. Today, there are many compounds of calcium phosphate with different calcium and phosphate ion availabilities [7]. Investigating the effect of TCP on demineralization, Amaechi et al. [23] concluded that TCP containing urea and silica was more effective in preventing demineralization compared to 225 ppm fluoride application and control groups. Also, in the* in vitro* study of Sri-Aulawarat et al. [24], ACP varnish was compared with TCP varnish and the latter group showed more reduction in demineralization, which is consistent with the present results.

On the other hand, Rirattanapong et al. [11] found no statistically significant difference between ACP, TCP, and varying concentrations of fluoride varnishes in terms of inhibiting demineralization. This may be due to the effect of artificial saliva on hardening the enamel surface, as well as nonstandardization of the measuring point on enamel surface. Furthermore, the outcomes of the present study were not consistent with that of Schemehorn et al. [7], who reported more fluoride uptake with the ACP varnish formulation. This difference might be attributed to the use of bovine teeth, different effects of artificial saliva on the availability of calcium and phosphate ions in ACP and TCP formulations, or measurement of the fluoride amount only on the cavity surface, while the fluoride in the medium is removed [7].

There are many salts of calcium phosphate with different calcium and phosphate ion availabilities. Among these, ACP is noncrystalline and has no systematic structure, thereby making it more soluble and more reactive than other crystalline calcium phosphates. ACP dissolves quickly and provides fast apatite reprecipitation for a demineralized lesion [25]. In the oral environment, ACP is unstable because the calcium and phosphate are delivered separately, which enables the precipitation of ACP on the tooth surface. On the other hand, TCP is a fairly insoluble crystalline form of calcium phosphate and is similar to apatitic calcium phosphate in tooth enamel [7]. When TCP comes into contact with the tooth surface and combines with saliva, the protective barrier breaks down making calcium and phosphate available [11]. The difference between the results of TCP and ACP groups in the present study might be attributed to different rates of availabilities of calcium and phosphate ions throughout the 14-day pH cycling period.

An increase in the acid resistance of dental enamel after Er:YAG laser irradiation has been proposed in several studies [12, 13, 26]. However, there are conflicting results regarding the effect of Er:YAG laser regarding the decrease of enamel solubility [13]. Liu et al. [27] assessed the optimal laser energy range between 100 and 200 mJ for the laser induced caries prevention with Er:YAG laser without water cooling and concluded that caries prevention might be achieved by using Er:YAG laser if the optimal ranges of laser parameters were chosen. In contrast to Liu et al., in the present study 80 mJ irradiation with 200 *μ*s pulse duration was used and it was found ineffective against enamel demineralization. This result might be related to the difference between the energy output and pulsed character of Er:YAG laser that may leave nonlased areas inadvertently between pulses.

In addition to power settings, application techniques also play an important role in the outcome of laser treatments. Cecchini et al. [13] evaluated alterations occurring after Er:YAG laser irradiation with different parameters. They compared hand tools in varying wavelengths that are either in contact with the enamel surface or not and concluded that when low wavelengths are applied in noncontact mode, Er:YAG lasers could proceed prohibition of demineralization. However, to ensure the consistent spot size with hand irradiation, an endodontic file was fixed at the handpiece and laser was applied from 12 mm distance. In the present study, laser was applied similar to the study of Correa-Afonso et al. [28], who indicated that Er:YAG laser was efficient in preventing demineralization at a 4 mm distance using water cooling. Even though the same parameters were used, the difference between the methods of analysis limits comparison of the results. In the present study, surface microhardness analysis gave information about the solidity of the enamel, whereas SEM analysis revealed the surface topography of the enamel after the application of different treatment modalities. Factors such as the application technique, method of analysis, and homogeneity of the irradiated area can influence the outcomes of the study, which might explain the variations between the results.

The use of water cooling is usually recommended to prevent overheating the surface of the enamel and causing ablations. Rodríguez-Vilchis et al. [29] and Hossain et al. [30] reported that a caries prevention effect was less significant when water cooling was used. Hossain et al. [30] used wavelength fixed at 2.94 *μ*m energy output from 100 to 150 mJ and pulse repetition rate of 10 Hz in different parameters in three study groups and observed similar craters in SEM analysis as seen in the present study. Hossain et al. [30] used Er:YAG laser at 400 mJ pulse energy with or without water cooling and reported that, without water cooling, the lased areas were melted and thermally degenerated. These results might be possibly explained by the study of Correa-Afonso et al. [28], where they could not find a satisfactory caries prevention at the analyzed surfaces due to the lack of heating required for chemical and ultrastructural alterations.

Similar to the present study, Apel et al. [31] preferred to use Er:YAG laser under natural conditions to better replicate a clinical situation and concluded that subablative energy densities of Er:YAG laser could not be recommended for caries prevention in clinical usage. Their data showed loss of hardness on the enamel surface, which is important for demineralization because of the resultant cracks, which are prone to accumulation of bacteria.

Further* in vivo* and* in situ* studies are required to better explain the effects of different varnish formulations and different Er:YAG laser parameters on caries prevention.

## 5. Conclusions

Within the limitations of this* in vitro* study, it was concluded that TCP varnish enhanced remineralization around brackets more effectively than ACP varnish. Nevertheless, both varnishes were found effective against demineralization around orthodontic brackets and therefore might be preferred for preventive treatments in orthodontic patients, with fixed orthodontic appliances. Inhibition of demineralization by Er:YAG laser irradiation as described in this study was found to be ineffective.

## Figures and Tables

**Table 1:** Multiple comparisons of microhardness values of specimens from 3 positions and 5 depths from the coronal side of bracket surface.

Distances from bracket surface	Depths	Er:YAG laser group		Control group		TCP group		ACP group		*P*
		Mean	SD	Mean	SD	Mean	SD	Mean	SD	
0 *μ*m	10 *μ*m	331.31	25.64	323.81	31.48	362.83	37.95	351.42	24.60	0.009*
	20 *μ*m	335.90	26.42	323.91	20.89	378.53	23.12	355.36	20.47	0.000*
	40 *μ*m	322.92	25.55	312.21	22.59	373.44	12.85	355.17	24.90	0.000*
	70 *μ*m	306.69	22.51	291.20	26.20	356.37	16.32	330.87	25.43	0.000*
	90 *μ*m	283.63	21.76	286.20	15.96	345.10	19.68	321.30	23.83	0.000*

100 *μ*m	10 *μ*m	358.05	14.52	350.14	17.76	393.68	11.28	364.23	6.15	0.000*
	20 *μ*m	359.67	14.78	348.88	29.11	392.84	10.88	370.85	7.64	0.000*
	40 *μ*m	347.35	16.69	338.79	23.86	380.27	10.51	358.81	22.92	0.000*
	70 *μ*m	327.77	17.69	313.54	31.97	367.99	11.41	329.78	25.81	0.000*
	90 *μ*m	311.72	23.71	302.04	31.74	352.48	12.13	318.90	24.99	0.000*

200 *μ*m	10 *μ*m	361.07	10.87	331.81	30.32	392.05	10.54	356.75	15.67	0.000*
	20 *μ*m	356.66	13.52	338.37	16.48	385.45	13.96	353.79	18.20	0.000*
	40 *μ*m	668.70	1042.84	320.36	30.06	367.85	12.02	348.08	18.67	0.002*
	70 *μ*m	315.02	20.18	294.23	30.15	357.53	11.86	334.81	18.65	0.000*
	90 *μ*m	289.22	34.87	276.30	28.59	344.01	13.13	318.49	24.44	0.000*

*P < 0.05: significance level; SD: standard deviation.

**Table 2:** Post hoc statistical comparisons of microhardness values of specimens from 3 positions and 5 depths from the coronal side of bracket surface.

Distances from bracket surface	0 *μ*m					100 *μ*m					200 *μ*m				
Depths	10 *μ*m	20 *μ*m	40 *μ*m	70 *μ*m	90 *μ*m	10 *μ*m	20 *μ*m	40 *μ*m	70 *μ*m	90 *μ*m	10 *μ*m	20 *μ*m	40 *μ*m	70 *μ*m	90 *μ*m
Laser/control	0.733	0.199	0.256	0.112	0.820	0.241	0.520	0.545	0.307	0.45	0.051	0.052	0.070	0.096	0.257
Laser/TCP	0.013	0.002*	0.000*	0.000*	0.000*	0.001*	0.000*	0.001*	0.000*	0.001*	0.000*	0.001*	0.006*	0.000*	0.001*
Laser/ACP	0.069	0.031	0.008*	0.028	0.002*	0.570	0.570	0.071	0.734	0.364	0.571	0.705	0.405	0.045	0.034
Control/TCP	0.008*	0.001*	0.000*	0.000*	0.000*	0.000*	0.000*	0.001*	0.000*	0.000*	0.000*	0.000*	0.001*	0.000*	0.000*
Control/ACP	0.059	0.008*	0.003*	0.006*	0.003*	0.017	0.019	0.049	0.151	0.199	0.070	0.059	0.082	0.004*	0.003*
TCP/ACP	0.096	0.005*	0.041	0.013	0.026	0.001*	0.000*	0.006*	0.001*	0.001*	0.000*	0.001*	0.014	0.004*	0.005*

*P < 0.005: significance level.

## References

[1] Tanna N, Kao E, Gladwin M, Ngan PW (2009). Effects of sealant and self-etching primer on enamel decalcification—part I: an *in-vitro* study. *The American Journal of Orthodontics and Dentofacial Orthopedics*.

[2] Behnan SM, Arruda AO, González-Cabezas C, Sohn W, Peters MC (2010). *In-vitro* evaluation of various treatments to prevent demineralization next to orthodontic brackets. *The American Journal of Orthodontics and Dentofacial Orthopedics*.

[3] Hughes DO, Hembree JH, Weber FN (1979). Preparations to prevent enamel decalcifications during orthodontic treatment compared. An *in vitro* study. *The American Journal of Orthodontics*.

[4] Santos LDM, dos Reis JIL, de Medeiros MP, Ramos SM, de Araújo JM (2009). *In vitro* evaluation of fluoride products in the development of carious lesions in deciduous teeth. *Brazilian Oral Research*.

[5] Tenuta LMA, Cury JA (2010). Fluoride: its role in dentistry. *Brazilian Oral Research*.

[6] Seppä L, Leppänen T, Hausen H (1995). Fluoride varnish versus acidulated phosphate fluoride gel: a 3-year clinical trial. *Caries Research*.

[7] Schemehorn BR, Wood GD, McHale W, Winston AE (2011). Comparison of fluoride uptake into tooth enamel from two fluoride varnishes containing different calcium phosphate sources. *Journal of Clinical Dentistry*.

[8] Rose RK (2000). Effects of an anticariogenic casein phosphopeptide on calcium diffusion in streptococcal model dental plaques. *Archives of Oral Biology*.

[9] Reynolds EC (1997). Remineralization of enamel subsurface lesions by casein phosphopeptide-stabilized calcium phosphate solutions. *Journal of Dental Research*.

[10] Skrtic D, Hailer AW, Takagi S, Antonucci JM, Eanes ED (1996). Quantitative assessment of the efficacy of amorphous calcium phosphate/methacrylate composites in remineralizing caries-like lesions artificially produced in bovine enamel. *Journal of Dental Research*.

[11] Rirattanapong P, Vongsavan K, Tepvichaisillapakul M (2011). Effect of five different dental products on surface hardness of enamel exposed to chlorinated water *in vitro*. *Southeast Asian Journal of Tropical Medicine and Public Health*.

[12] Liu Y, Hsu C-YS, Teo CMJ, Teoh SH (2013). Subablative Er:YAG laser effect on enamel demineralization. *Caries Research*.

[13] Cecchini RCM, Zezell DM, de Oliveira E, de Freitas PM, Eduardo CDP (2005). Effect of Er:YAG laser on enamel acid resistance: morphological and atomic spectrometry analysis. *Lasers in Surgery and Medicine*.

[14] Hsu C-YS, Jordan TH, Dederich DN, Wefel JS (2000). Effects of low-energy CO_2_ laser irradiation and the organic matrix on inhibition of enamel demineralization. *Journal of Dental Research*.

[15] Fried D, Giena R, Featherstone JD, Seka W, Wigdor HA, Featherstone JDB, White JM Multiple pulse irradiation of dental hard tissues at CO2 laser wavelengths.

[16] Hu W, Featherstone JDB (2005). Prevention of enamel demineralization: an *in-vitro* study using light-cured filled sealant. *The American Journal of Orthodontics and Dentofacial Orthopedics*.

[17] Murray JJ, Nunn JH, Steele JG (2003). *The Prevention of Oral Disease*.

[18] Staudt CB, Lussi A, Jacquet J, Kiliaridis S (2004). White spot lesions around brackets: *in vitro* detection by laser fluorescence. *European Journal of Oral Sciences*.

[19] Reynolds EC (2008). Calcium phosphate-based remineralization systems: scientific evidence?. *Australian Dental Journal*.

[20] Uysal T, Amasyali M, Koyuturk AE, Ozcan S (2010). Effects of different topical agents on enamel demineralization around orthodontic brackets: an in vivo and *in vitro* study. *Australian Dental Journal*.

[21] Hong YC, Chow LC, Brown WE (1985). Enhanced fluoride uptake from mouthrinses. *Journal of Dental Research*.

[22] Chow LC, Guo MK, Hsieh CC, Hong YC (1981). Apatitic fluoride increase in enamel from a topical treatment involving intermediate CaHPO_4_·2H_2_O formation, an in vivo study. *Caries Research*.

[23] Amaechi BT, Karthikeyan R, Mensinkai PK, Najibfard K, MacKey AC, Karlinsey RL (2010). Remineralization of eroded enamel by a NaF rinse containing a novel calcium phosphate agent in an in situ model: a pilot study. *Clinical, Cosmetic and Investigational Dentistry*.

[24] Sri-Aulawarat W, Nakornchai S, Thaweboon S, Korsuwannawong S (2012). Effect of tricalcium phosphate, casein phosphopeptide-amorphous calcium phosphate and sodium fluoride products on demineralization of artificial advanced enamel lesions. *International Journal of Oral Research*.

[25] Dorozhkin SV (2010). Amorphous calcium (ortho)phosphates. *Acta Biomaterialia*.

[26] Apel C, Meister J, Götz H, Duschner H, Gutknecht N (2005). Structural changes in human dental enamel after subablative erbium laser irradiation and its potential use for caries prevention. *Caries Research*.

[27] Liu JF, Liu Y, Stephen HCY (2006). Optimal Er:YAG laser energy for preventing enamel demineralization. *Journal of Dentistry*.

[28] Correa-Afonso AM, Ciconne-Nogueira JC, Pécora JD, Palma-Dibb RG (2012). *In vitro* assessment of laser efficiency for caries prevention in pits and fissures. *Microscopy Research and Technique*.

[29] Rodríguez-Vilchis LE, Contreras-Bulnes R, Sánchez-Flores I, Samano EC (2010). Acid resistance and structural changes of human dental enamel treated with Er:YAG laser. *Photomedicine and Laser Surgery*.

[30] Hossain M, Nakamura Y, Kimura Y, Yamada Y, Ito M, Matsumoto K (2000). Caries-preventive effect of Er:YAG laser irradiation with or without water mist. *Journal of Clinical Laser Medicine and Surgery*.

[31] Apel C, Birker L, Meister J, Weiss C, Gutknecht N (2004). The caries-preventive potential of subablative Er:YAG and Er:YSGG laser radiation in an intraoral model: a pilot study. *Photomedicine and Laser Surgery*.

